# A randomised controlled trial evaluating the Guide Cymru mental health literacy intervention programme in year 9 (age 13–14) school pupils in Wales

**DOI:** 10.1186/s12889-023-15922-2

**Published:** 2023-06-05

**Authors:** Nicola J. Simkiss, Nicola S. Gray, Andrew H. Kemp, Chris Dunne, Robert J. Snowden

**Affiliations:** 1 Department of Psychology, School of Psychology, Swansea University, SwanseaWales, SA2 8PP UK; 2 Caswell Clinic, Swansea Bay University Health Board, Bridgend, UK; 3 Action for Children, Head Office, 3 The Boulevard, Ascot Road, Watford, UK; 4 School of Psychology, Cardiff University, Cardiff, UK

**Keywords:** The Guide, Mental Health, Adolescents, Mental health literacy, Mental health stigma, Help-seeking Behaviour, Avoidant coping

## Abstract

**Background:**

Adolescent mental health has become a public health concern as 10–20% of adolescents have experiences with mental health problems. Improving mental health education is critical to reducing stigma and improving access to appropriate care when needed. Here we examine the impact of a mental health literacy programme (Guide Cymru) in young adolescents in the UK. A randomised controlled trial assessed the effectiveness of the Guide Cymru intervention.

**Method:**

A total of 1,926 pupils (860 males and 1066 females) aged 13–14 (year 9) took part in the study. The secondary schools were randomised into the active and control arms of the study. Teachers in the active arm of the study were trained on the Guide Cymru and then delivered the intervention to their pupils. Pupils in the active groups received six modules of mental health literacy (the Guide Cymru), and control schools received teaching as usual. Mental health literacy across several domains (e.g., knowledge, stigma, help-seeking intentions) were assessed both before and after the intervention. Data collection for the randomised controlled trial ran from September 2019 to March 2020. Multi-level modelling analysis was conducted to account for the clustered nature of the design.

**Results:**

All aspects of mental health literacy, including mental health knowledge (*g* = 0.32), good mental health behaviours (*g* = 0.22), mental health stigmas (*g* = 0.16), intentions to seek help (*g* = 0.15), and avoidant coping (*g* = 0.14) improved after completing the Guide Cymru programme (*p*s < .001).

**Discussion:**

The current study presents evidence for the Guide Cymru’s effectiveness in improving secondary school pupils' mental health literacy. We demonstrate that providing teachers with appropriate resources and training to deliver the Guide Cymru programme within their classrooms can improve the mental health literacy of pupils. These findings have important implications for the beneficial impacts the secondary school system can have on reducing the burden of mental health problems at a critical point in a young person’s life.

**Trial registration:**

ISRCTN15462041. Registered 03/10/2019.

## Background

Adolescent mental health has received growing attention recently and has become a public health concern [1]. Worldwide, approximately 10–20% of adolescents have had experiences with mental health problems [2]. In Wales alone, the specialist Child and Adolescent Mental Health Service in Wales (CAMHS) is under more pressure as the last four years have seen a significant increase in demand [3]. A recent survey conducted by Page, Hewitt [4] in Wales, identified that almost 2 in 5 (39%) young people reported mental health symptoms, with almost 1 in 5 (19%) reporting ‘very high’ mental health symptoms as reported on the Strengths and Difficulties Questionnaire [5]. Poor mental health during adolescence can affect individuals’ well-being, functioning and development [6]. Left untreated, this can extend into adulthood, resulting in both physical and mental health problems [2]. Although mental health problems are prevalent among adolescents, seeking appropriate help for such issues remains low [7]. Here, we report on the results of a randomised controlled trial to assess the effectiveness of a mental health literacy programme (the Guide Cymru) designed to improve mental health literacy in young adolescents (year 9 students, age 13–14 years). A protocol for the study, including its rationale, was previously published [8].

With increased independence and the development of decision-making abilities, adolescence is a crucial period for learning and adopting health-promoting behaviours [9]. Health literacy is a person's ability to understand and use information appropriately to make decisions about their health**,** which is key to improving health outcomes at individual and population levels [1]. Mental health literacy, a component of health literacy, can be defined as “*knowledge and beliefs about mental disorders which aid in the management, recognition, and prevention*” [10]. Identifying a mental health problem is the first step to obtaining help and support, and mental health literacy (MHL) aids in this initial step [11]. MHL is known to consist of several components, for example, (a) the ability to identify and recognise a mental health disorder, (b) an understanding of the risk factors and causes of mental health disorders, (c) understanding and knowledge about help-seeking behaviours, [10]. A systematic review by Gulliver et al. [8] identified how poor mental health literacy was one of the most critical barriers to seeking appropriate support for a mental health problem amongst adolescents [12].

Mental health literacy programmes aim to improve knowledge about mental health and reduce the stigma that is often associated with poor mental health. Further to this, such programmes aim to prevent further development of mental health conditions and improve and encourage seeking formal / specialist mental health support. Given the young age of onset for many mental health problems [13], it may seem prudent to deliver such programmes to people at a young age. Schools are ideally placed to do this as they can deliver such programmes in a sustainable manner [12] and can reach all pupils rather than those that seek out such programmes due to their own mental health problems. Educating all pupils in mental health literacy aims to reduce mental health related stigma towards people with mental health problems, and may, in turn, help reduce self-stigma in those that do have mental health problems. Furthermore, having a robust and influential figure such as a teacher educating about mental health can help build young people's confidence to speak about mental health to someone they trust [14]. Promoting young people’s mental health within the school environment can also have beneficial educational impacts [15].

Growing evidence suggests that early school-based mental health interventions often demonstrate significant benefits of reduced mental health stigma and improved mental health knowledge [16, 17]. Although, evidence-based educational interventions are limited for adolescents and young people [18], a recent review by Yan et al. [19] found moderate evidence for school-based mental health interventions in the effectiveness of improving mental health literacy and reducing mental health stigma. Despite the need for mental health promotion within secondary schools, there are several common barriers which include funding and limited staff capacity [20]. Furthermore, recent reviews of mental health programmes for secondary school teachers suggest that more evidence is needed to understand ways to support teachers in improving their helping behaviours [21]. Research has often identified that teachers' knowledge of mental health is limited. Teachers often do not feel confident in delivering a mental health programme and would benefit from appropriate education and training [22].

### The Guide Cymru

The Guide Cymru is a mental health literacy programme by Action for Children (a UK-based charity based on The Guide [19]. It is focused on young adolescents (13 to 14 years) and has both in English and Welsh language versions. The Guide Cymru comprises six modules: understanding mental health and mental illness, stigma myths and realities, information on specific mental illnesses, experiences of mental illness, help-seeking and finding support, and the importance of positive mental health.

The Mental Health & High School Curriculum Guide (or The Guide) is a school-based mental health literacy resource developed by mental health experts, educators and the Canadian Mental Health Organisation [23]. The Guide has demonstrated sustained improvements in students’ mental health literacy in Canadian schools [24]. Milin et al. [25] examined the efficacy of the Guide in a randomised controlled trial on 534 students (362 in active arm, 172 in control arm) in grade 11 and 12 (age 16–17 years) pupils in Canada. The Guide improved mental health knowledge and produced more positive attitudes towards mental health problems (reduced stigma). However, these improvements occurred only in students in the “university” stream and were not apparent in those in the “community college” stream. This might suggest that only the more able students benefitted from the Guide. Hence, students of a lower age may not benefit from the Guide. Hence, in developing the Guide Cymru professionals were consulted with respect to the content so as to be understandable by most students in year 9 (age 13–14) in the UK.

### Aims and hypothesis

This clustered randomised controlled trial evaluated the effectiveness of the intervention. Members of the Action for Children team delivered training on The Guide Cymru to teachers, who then delivered the content to a cohort of Year 9 pupils aged between 13 and 14 in secondary schools across Wales. We aimed to administer the Guide Cymru to a younger age group (13–14 years) compared to Milin et al.’s [25] sample as research has often identified that half of all lifetime diagnosable mental health disorders begin by 14 years [13]. This involved a process of re-writing The Guide to be appropriate for a Welsh Context, simplifying terminology surrounding mental health and mental health problems, providing additional resources for lower-ability pupils and access to relevant video clips that complemented the materials. All materials were available for schools to support all backgrounds. Following this, two age-appropriate children reviewed the questions to help to understand the clarity and accuracy of wording; they also provided feedback on the content and relevance of each question.

A recent review identified a lack of psychometrically sound mental health literacy scales for use in children and adolescents, which highlights a significant limitation in the current methods of child focused mental health literacy and evaluation of programmes [26]. The Knowledge and Attitudes to Mental Health Scales (KAMHS; [27]) was developed for this study and was designed to capture the potential impact of the Guide Cymru. KAMHS has been found to have good psychometric properties [27].

We hypothesised that children that received the Guide Cymru would show greater mental health literacy as indicated by: (1) greater knowledge of mental health and mental health problems, (2) reduced stigma towards people with mental health problems, (3) reduced levels of self-stigma, (4) a greater intention to seek help if they have a mental health problem, and (5) better mental health-related behaviours.

## Method

### Participants

**Fig. 1:**
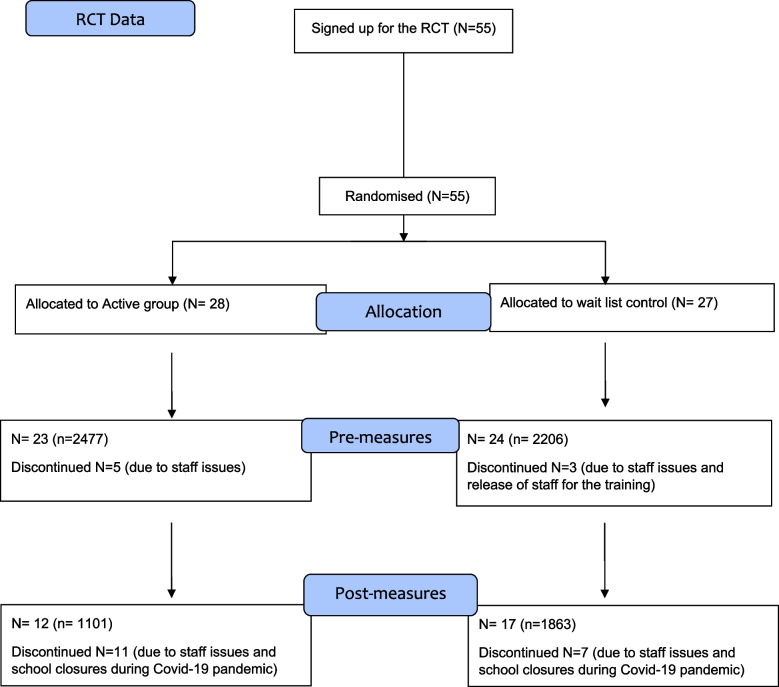
CONSORT diagram demonstrating the flow of participants through each stage of the Randomised Controlled Trial

All 205 public secondary schools in Wales were invited to participate in the research study. A total of 29 schools successfully took part in the trial. Figure 1 shows a CONSORT statement of the procedures and participant numbers included in the randomised controlled trial.

There were several reasons for schools not completing the planned programme delivery and data collection. These included the practicality of delivering the programme within 12 weeks, schools being unable to release teaching staff for training, and school closures during the Covid-19 pandemic. In total 860 males and 1066 females took part in the trial.

A power analysis was conducted to determine the appropriate sample size for the research study (see [8]). Our original calculation, before the Covid-19 pandemic, calculated that a normal (non-clustered) randomised controlled trial power analysis with parameters of alpha = 0.05, power of 80%, and a standardised effect size of 0.30 (small effect size for Cohen’s *d* and close to that obtained by Milin, Kutcher [25] required 175 pupils per group (N = 350). To account for clustering, we estimated that our average cluster size would be around 150 pupils per school and when coupled with an intra-cluster correlation of 0.10 leads to a design effect of 16. Therefore, our initial aim was to have a sample size of 2800 per group. In March 2020, the closure of secondary schools due to Covid-19 curtailed the data we were able to collect. However, research has highlighted that intra-cluster correlation effects are generally 0.01–0.02 in human studies [28] and so our initial estimate was too conservative. Multi-level modelling was used to calculate the actual intra-cluster correlation in the collected data. Intra-cluster correlations ranged from 0.003 to 0.05 (mean = 0.02, SD = 0.01). Using this intra-cluster correlation of 0.02 produces a design effect of 4, so the sample size needed was 700 (175*4) participants per group. Hence, the study achieved the required sample size and was adequately powered.

### Design

The evaluation of the Guide Cymru used a two-armed cluster randomised controlled trial. A cluster-design was necessary as The Guide Cymru programme was delivered to the pupils by their teachers. Therefore, the school was the unit of clustering. Further details can be found in the study protocol [8]. The study had a mixed effects design with a within-subjects factor of time (baseline and post-intervention) and a between-subjects factor of intervention (the Guide Cymru vs control). Schools that consented and agreed to take part in the research study were randomised into either the control arm which continued teaching as usual, or the intervention arm which received the 12-week intervention programme (The Guide Cymru).

Schools were stratified by geographic location to facilitate the researchers visiting the schools. For each location (typically consisting of 6 -12 schools) each school was randomised into either the active or control arm of the study using a computer-generated random sampling procedure to ensure unbiased allocation to each group prior to data collection: (https://www.random.org/). Following this, schools were then yoked. The yoking process involved allocating an intervention school to a control school according to geographical location, as this helped keep the demographic make-up of the active and control groups similar and was beneficial for data collection as the two yoked schools could be visited on the same day.

### Setting

Data collection for the study ran from 1^st^ September 2019 to 30^th^ March 2020. Fifty-five secondary schools originally signed up to participate in the trial, and 47 schools took part in baseline measures. However, due to the onset of restrictions due to the Covid-19 pandemic, only 29 secondary schools were able to provide both baseline and post-intervention data.

### Procedure

Baseline mental health literacy measures were collected from both active and control schools. This wave occurred approximately one week before the schoolteachers in the active group were trained on administering the Guide Cymru. The ‘Go-To’ Guide training involves a two-day teaching session for teachers to become familiar with the materials and improve their own mental health training. Teachers would then deliver the intervention programme to their Year 9 pupils over the following 10 to 12 weeks. The control school was tested the same day as their yoked active school.

Wave two occurred 1–2 weeks after the delivery of the Guide Cymru for the students in the active arm of the study. The control school(s) was evaluated on the same day as their yoked active school(s). Data was due to be collected in a third wave at 12 weeks post intervention. However, due to the closure of secondary schools to the Covid-19 pandemic this third wave of data could not be collected. Data collection took place face to face during lesson time within each secondary school. The PhD student and a member of staff from Action for Children completed data collection.

### Intervention

Active schools received The Guide Cymru, an intervention aimed at improving knowledge and attitudes towards mental health in adolescent populations. Based on The Guide [23], which was initially written for and tested on 16–17 -year-olds in North America, the Guide Cymru includes six modules to mental health: Understanding Mental Health and Mental Illness, Seeking help and the importance of positive mental health. The Guide Cymru consists of a two-day training course for teachers (termed the Go-To Educator) that covers the materials in the Guide Cymru and the learning resources available. These teachers then delivered the Guide Cymru to their students over the following 10–12 weeks. The control group pupils received teaching as usual.

### Outcome measures

#### Knowledge and Attitudes to Mental Health Scales (KAMHS) [27]

The KAMHS measure is a questionnaire designed for children and adolescents aged 11–16 years measuring mental health literacy over six domains: (1) Knowledge, (2) Good mental health behaviours, (3) (Lack of) Stigma to others, (4) (Lack of) Self-stigma, (5) (Lack of) Avoidant coping, and (6) Help-seeking behaviours. Scales 3–5 were called “lack of” so that higher scores on all sub-scales are indicative of higher mental health literacy. The KAMHS also contains a social desirability scale to measure and control for positive impression management. Participants were asked to respond to statements on a five-point Likert scale (strongly agree, agree, don’t know, disagree, strongly disagree) with higher scores indicating higher mental health literacy (e.g., greater knowledge, less stigma, greater intentions to seek help, etc.). The KAMHS has been shown to have good psychometric properties [27].

### Ethical approval

Completed questionnaires contained limited personal information to preserve anonymity. Gender and age were the only demographics collected from pupils. The trial received full ethical approval by Swansea University Ethical Committee on 29/10/2019 (Reference number: 2018–0272–259). Informed consent from participants was waived by the Swansea University Ethical Committee. This was on the basis that the intervention programme was being delivered as part of the National Curriculum and was being delivered within schools alongside other lessons within the learning curriculum. Parents were informed by each school that the Mental Health Literacy programme was being delivered as part of the curriculum and were given the opportunity to withdraw their child from these lessons if they wished.

### Matching of participant data

In line with Kutcher, Wei [29], pre and post-questionnaires were matched via four “code” questions that track the person pre- and post-completion of the programme. These were: “name of first pet”, “date of your birthday (e.g., 23rd)”, “house number”, and “favourite colour”.

### Data analysis

Multilevel Modelling (MLM), conducted via SPSS Version 26, was used to analyse the data with a mixed design to evaluate the effectiveness of the Guide intervention by examining change over time (from pre-intervention to post-intervention) in the two groups (intervention vs control). School was entered as a random clustering effect for all analyses. Effects sizes for change in scores on the KAMHS subscales due to the intervention (and for the control group) were calculated (Hedges G). Missing responses were pro-rated to the scale mean. If more than 50% of the items were missing from the questionnaires, they were excluded from the analysis. A minimum of 10% veracity checks were completed on all data entries. Due to the nature of the research, the researcher was not blinded during the analysis.

## Results

Mean and standard errors for intervention and control groups for pre-and post-intervention are shown in Table 1. A preliminary analysis compared the scores from each of the subscales between participants in the active and control arms of the study at baseline (pre-intervention). Independent t-tests revealed no significant differences on any of the scales (all *p*s > 0.47, all *d*s < 0.03). Hence, the two arms of the study were well matched in terms of baseline levels of mental health literacy.

**Table 1:** Mean and standard errors (in brackets) for the intervention and control groups for pre- and post-intervention. Higher scores indicate a better mental health literacy score, with a possible range of 0–4

KAMHS subscale	Intervention		Control	
	Pre	Post	Pre	Post
Knowledge	2.24 (.01)	2.35 (.01)**	2.25 (.02)	2.25 (.01)
Good mental health behaviours	2.65 (.02)	2.77 (.02)**	2.64 (.01)	2.67 (.02)
(Lack of) Stigma to others	2.98 (.02)	3.08 (.02)**	3.00 (.02)	2.95 (.02)┼
(Lack of) **S**elf-stigma	2.33 (.03)	2.38 (.03)**	2.33 (.02)	2.36 (.02)**
(Lack of) Avoidant coping	2.30 (.02)	2.39 (.02)**	2.28 (.02)	2.30 (.02)
Help-seeking behaviours	2.35 (.03)	2.47 (.03)**	2.37 (.02)	2.36 (.02)

^**^increase in score pre to post *p* < .001. ^┼^ decrease in score pre to post *p* < .001

### Knowledge

Multilevel modelling (MLM) analysis revealed a significant main effect of time *F*(1, 1939.77) = 64.37, *p* < 0.001, and a significant main effect of condition, *F*(1, 1957) = 11.13, *p* = *0.0*01. There was a significant time-by-intervention effect on pupils’ knowledge, *F*(1, 1941) = 47.80, *p* < 0.001.

Pairwise comparisons of the active group showed a significant increase in knowledge scores from pre to post intervention, *t*(755) = 8.22,* p* < 0.001, *g* = 0.32, 95% CI [0.24,0.40]. There was no significant change for the control pupils from pre to post, *t*(1183) = -1.03, *p* = 0.31, *g* = 0.03, 95% CI [-0.02, 0.09].

### Good mental health behaviours

MLM analysis revealed a significant main effect of time *F*(1, 1939) = 37.09, *p* < 0.001, and a significant main effect of intervention, *F*(1, 1957.27) = 8.34, *p* = 0.004. There was also a significant time-by-intervention effect on good mental health behaviours *F*(1, 1941) = 14.58, *p* < 0.001.

Pairwise comparisons of the active group showed a significant increase in good mental health behaviour scores from pre to post intervention, *t*(755) = 5.98, *p* < 0.001, *g* = 0.22, CI 95% [0.15, 0.30]. Control pupils did not show an increase in scores across time, *t*(1183) = 1.96, *p* = 0.05, *g* = 0.06, 95% CI [0.002, 0.12].

#### (Lack of) Stigma to others

MLM analysis revealed a non-significant main effect of time *F*(1, 1936) = 3.69, *p* = 0.055, but a significant main effect of condition, *F*(1, 1961) = 5.27, *p* = 0.022. There was a significant time-by-intervention effect *F*(1, 1938) = 36.39, *p* < 0.001.

Pairwise comparisons of the active group showed a significant increase in a Lack of Stigma to others scores (or, in other words, a decrease in stigma to others) from pre to post intervention *t*(755) = 4.72, *p* < 0.001, *g* = 0.16, 95% CI [0.09, 0.23]. Whereas control pupils showed a significant decrease from pre to post, *t*(1183) = -3.56, *p* < 0.001, *g* = -0.09, 95% CI [-0.03, -0.14].

#### (Lack of) Self-stigma

MLM analysis revealed a significant main effect of time *F*(1, 1939) = 6.12, *p* = 0.01, but no main effect of condition* F*(1, 1964) = 0.14, *p* = 0.71. There was no significant interaction of time-by-intervention on pupils’ lack of self-stigma, *F*(1, 1941) = 0.29, *p* = 0.59.

#### (Lack of) Avoidant coping

MLM analysis revealed a significant main effect of time *F*(1, 1939) = 14.08, *p* < 0.001, and a significant main effect of condition* F*(1, 1957.81) = 3.84, *p* = 0.050. There was a significant time-by-intervention effect on pupils' Lack of Avoidant coping score, *F*(1, 1941) = 5.02, *p* = 0.03.

Pairwise comparisons of the active group showed a significant increase in a Lack of Avoidant coping scores (or, in other words, a decrease in avoidance coping) from pre (*M* = 2.30, *SE* = 0.02) to post intervention, *t*(755) = 3.67, *p* < 0.001, *g* = 0.14, 95% CI [0.06, 0.21]. Control pupils showed no significant difference pre to post intervention (*M* = 2.30, *SE* = 0.02), *t*(1183) = 1.37, *p* = 0.18, *g* = 0.03, 95% CI [-0.03, 0.09].

#### Help-seeking behaviours

MLM analysis revealed a significant main effect of time *F*(1, 1939) = 11.09, *p* = 0.001, but no significant main effect of condition, *F*(1, 1966.24) = 2.31, *p* = 0.13. There was a significant time-by-intervention effect on pupils’ intensions to seek help, *F*(1, 1941) = 17.08, *p* < 0.001.

Pairwise comparisons of the active group showed a significant increase in intentions to seek help from pre to post intervention, *t*(755) = 4.45, *p* < 0.001,* g* = 0.15, 95% CI [0.09, 0.22]. Control pupils' scores did not differ from pre to post, *t*(1183) = 0.78, *p* = 0.44, *g* = -0.03, 95% CI [-0.06, 0.03].

**Fig. 2:**
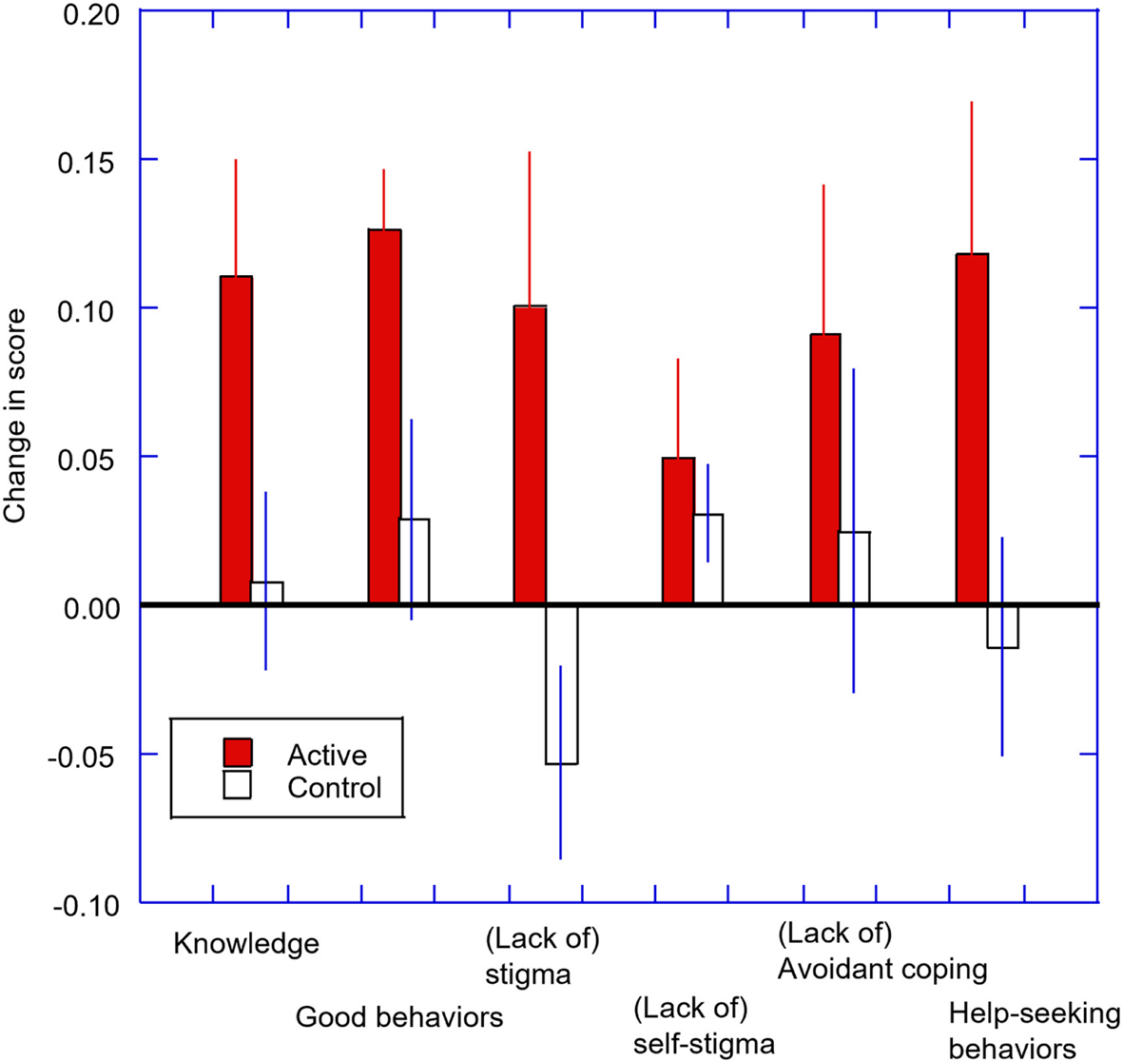
Change in scores (post–pre) for KAMHS subscales for active and control groups. Error bars indicate 95% confidence intervals

To summarise, Fig. 2 shows the change in scores from the pre-to-post intervention for each scale. Participants in the active arm showed improvement in nearly all the measures of mental health literacy, while those in the control group did not. The exception to this was for the (lack of) self-stigma scale where both groups showed improvement and so this improvement cannot be assigned to the Guide intervention.

## Discussion

The key findings of the randomised controlled trial were a significant improvement in mental health knowledge, mental health related behaviours, and help-seeking behaviours, with a reduction in stigmatic beliefs about people with mental health problems, in those pupils that received the Guide intervention in comparison to those that did not. We, therefore, demonstrate that providing teachers with appropriate resources and training to deliver the Guide Cymru programme within their classrooms can improve the mental health literacy of pupils.

These outcomes replicate the findings of Milin, Kutcher [25] but using a modified version of the Guide. The findings extend upon those of Milin et al. [25] as the Guide Cymru was administered to a younger age group (13–14 years) compared to Milin et al.’s [25] sample of 15–18 year-olds. Research has often identified that half of all lifetime diagnosable mental health disorders begin at 14 years or younger [13], so we targeted a younger age group to provide the appropriate resources to young people during this critical period. The present study demonstrates that the Guide Cymru successfully improves mental health literacy of this younger age group.

The study further extends the findings of Milin et al. [25] by using a more comprehensive assessment of mental health literacy. The study of Milin et al. [25] examined two aspects of mental health literacy: knowledge (about mental illness and the treatment of mental disorders), and stigma to others (negative attitudes and beliefs about people with mental health problems). In comparison, the present research extended this to show that the Guide Cymru was able to show pre-to post intervention improvements in good mental health behaviours, willingness to seek help, and avoidant coping styles. Self-stigma was also reduced post intervention, but this was also evident in the control group. We argue that while improvements in knowledge about mental health problems and a reduction in stigma towards others are important, it is these other aspects that may be more important in relationship to the person’s actual mental health. For instance, recent research has shown in an adult workforce that self-stigma, a lack of help-seeking, and avoidant coping were all associated with current mental health problems, whereas aspects of stigma towards people with mental health problems were not associated with current mental health problems [30]. An analysis of our data taken at Wave 1 (before any intervention for both groups) shows a highly similar pattern of results for our sample of young people [31]. We return to these points in the discussion below.

### Mental health knowledge

We demonstrate that the Guide Cymru significantly increased mental health knowledge from pre to post for intervention group participants compared to the control group. Research has often addressed how knowledge about mental health disorders may be crucial for early recognition of a mental health problem [7]. Once an individual can accurately identify a mental health problem, this may be the first step to receiving appropriate support [32]. Moreover, gaining knowledge about mental health has often demonstrated increased confidence and talking about your mental health. For example, research has reported changes in knowledge and attitudes surrounding mental health following a community conference about mental health [33]. Participants reported changes in knowledge regarding mental health issues, a more open attitude to discussing and discussing mental health, and greater acceptance of mental health issues in themselves and others.

### Good mental health behaviours

The Guide Cymru significantly increased pupils’ understanding of how to optimise and maintain good mental health from pre to post for intervention. Good mental health is a state of well-being that encourages individuals to be able to cope with the normal stresses of everyday life [34]. Therefore, the promotion of good mental health behaviours in adolescents and young people is an important way to promote good mental health at an early stage. Many health interventions have begun to promote the adoption of healthy habits that promote both mental and physical health [35]. Through the implementation of the Guide Cymru, schools can play an integral part in promoting good mental health behaviours.

#### (Lack of) Stigma to others

The findings demonstrate that the Guide Cymru can reduce people’s mental health stigma towards those that have mental health problems. These findings are consistent with the literature establishing the meaningful relationship between mental health knowledge and reducing stigma [36]. Over recent years, there has been growing evidence of mental health stigma amongst adolescents and young people [37] and so programmes that reduce such stigma are needed more than ever.

There appears to be no direct relationship between a person’s level stigma to others and their own mental health (at least in adults, see [25]). Indeed, it may even be that those with better mental health are more likely to show such stigmatic beliefs [26]. However, increased levels of stigma to others must, inevitably, be associated with greater levels of public stigma towards those with mental health problems. In turn, this will produce greater anticipated stigma and self-stigma in people with mental health problems. Hence, programmes that can decrease stigma to others should be welcomed even if there are no immediate benefits for the individual receiving such a programme.

#### (Lack of) Self-stigma

Participants that received the Guide Cymru showed an increase in scores on the Lack of Self-stigma scale (in other words they had less self-stigma related to mental health problems), although the magnitude of this effect appears smaller than for the other scales of the KAMHS (see Fig. 2). However, there was also an increase in scores in the control group, and so we were not able to demonstrate clear effects of the Guide Cymru for this variable. Given the strong relationship between self-stigma and mental health (and for help-seeking behaviours – see Yanos et al., [38], this may be an area for further development for this intervention programme to improve the power of the intervention to tackle self-stigma in young people, so that clear gains can be achieved.

#### (Lack of) Avoidant coping

The Guide Cymru improved avoidant coping among pupils. This should help pupils to deal with life stressors head-on and not avoid and ignore problems or concerns related to mental health problems. Stress is a pervasive feature of human development, particularly during adolescence [39]. Adolescents' response to such stressors can significantly predict how they adapt during this period [40]. For example, research has identified that adolescents who engage in more avoidant coping may be at a greater risk for poorer adjustment to life stressors [40]. Further, avoidance of stress is strongly linked to distress and depression, particularly among adolescents [41, 42]. Therefore, a reduction in avoidant coping amongst pupils should result in more appropriate help-seeking behaviour and better mental health.

#### Help-seeking behaviours

The Guide Cymru produced improvement in intentions to seek help for mental health problems. Rüsch, Evans-Lacko [43] also found that better mental health knowledge predicted stronger intentions to seek help and disclose a mental health problem to a friend or family member in an adult population. Future research may consider measuring actual mental health help-seeking behaviours instead of self-reported intentions, to see whether the Guide Cymru can produce behavioural change.

### Strengths and limitations

This study has numerous strengths. The study had strong statistical power provided by a large sample size and the protocol was preregistered and published ahead of data collection [44]. A further strength of the current study was using the KAMHS measurement tool. The KAMHS measure was specifically designed to capture the potential impact of the Guide Cymru and was developed to have good psychometric properties [27].

Despite the positive outcomes in improving pupils’ mental health literacy, this study is not without limitations. Follow-up of the Guide Cymru’s impact was taken only two weeks after the programme's completion, which is insufficient to evaluate whether the positive impact of the interventions was maintained over time. Unfortunately, the restrictions imposed due to the COVID-19 pandemic did not allow us to complete our intended longer-term repeat follow-up of our cohort in order to evaluate the degree to which positive changes in mental health literacy was sustained. Future research should replicate and extend the findings with longer follow-up periods. Future research may also consider reflecting upon pupil feedback in a process evaluation in their response to the programme. This may also be a consideration for looking at the sustainability of the Guide programme to see if further training is needed. Future research may also consider observing the potential impact on inequalities to assess generalisability and sustainability of the research going forward. It may also be beneficial to gather feedback from teachers on their perceptions of the training and the approaches they took to delivering the programme within their schools. This would be useful for understanding what mechanisms worked well and the sustainability of the programme.

The take-up rate of implementing the Guide Cymru from secondary schools was low (at 26.83%) despite the programme being offered without direct cost to the school. All secondary schools across Wales were invited to participate in the research, and several schools expressed interest. However, given the time restraints of the randomised controlled trial and the inability to release staff for training, many schools did not feel that they could implement the Guide. This particularly highlights how capacity of teachers to undertake the training of mental health literacy remains as an important barrier to these types of interventions. Both direct and indirect costs (e.g., staff time) need to be factored into research programmes attempting to intervene with child and adolescent mental health, and that funding streams should consider providing financial support for supply teachers and/or provide additional payments to teaching staff to undertake training during the weekends or school holidays. Further, the onset of the Covid-19 pandemic meant that many schools that were interested in implementing the Guide could not do so due to the restrictions imposed and school closures due to the Covid pandemic.

Finally, while many significant improvement in mental health literacy are demonstrated in this study, the magnitude of these changes were “small” in terms of effect sizes, though comparable with those found by Milin et al., [25]. It is as yet unclear how these small effects relating to mental health literacy might translate into changes in actual levels of mental health at either the individual or group level. Future studies measuring actual mental health, and over a far longer time period, are needed to address these issues.

## Conclusion

The Guide Cymru was able to show substantial improvement in the mental health literacy of adolescents (age 13–14) across a range of important areas. This included knowledge of mental health issues, and good mental health behaviours (including not using avoidant coping). It was also able to reduce levels of mental health related stigma and increased the intention to use help-seeking behaviours. These findings have important implications for the beneficial impact of the implementation of a school-based mental health literacy programme, in reducing the burden of mental health difficulties in adolescents and by teaching healthy coping at a critical developmental stage in a young person’s life. Along with the Welsh Government’s Mental Health and Wellbeing Strategy for Wales ‘Together for Mental Health’ [45], this research strongly aligns with the current aims of promoting mental well-being and preventing mental health problems.

We are hopeful that the Guide Cymru can be used and adapted in other countries to produce similar improvements in the mental health literacy of young people, with the objective that such improvements in knowledge, understanding, and reduced stigma about mental health will translate into better mental health outcomes for these young people for the rest of their lives.

## Data Availability

The datasets generated during and/or analysed following the current study are stored in a publicly available repository (Mendeley) (https://data.mendeley.com/datasets/tkb9z7b6jp/1). The data shared are an anonymised SPSS database that contains the item-by-item scores from the questionnaires as well as the scale scores and demographic information.
